# Ethyl 4-anilino-2,6-bis­(4-chloro­phen­yl)-1-phenyl-1,2,5,6-tetra­hydro­pyridine-3-carboxyl­ate

**DOI:** 10.1107/S1600536813013573

**Published:** 2013-05-22

**Authors:** Jianfeng Yu, Shiming Tang, Jingbin Zeng, Zifeng Yan

**Affiliations:** a Department of Chemistry, College of Science, China University of Petroleum, Qingdao 266555, People’s Republic of China; b State Key Laboratory of Heavy Oil Processing, China University of Petroleum, Qingdao 266555, People’s Republic of China

## Abstract

The title compound, C_32_H_28_Cl_2_N_2_O_2_, was synthesized by a multicomponent reaction of 4-chloro­benzaldehyde, aniline and ethyl aceto­acetate. The central 1,2,5,6-tetra­hydro­pyridine ring exhibits a distorted boat conformation and the two chloro­phenyl rings attached to the central ring at positions 2 and 6 are oriented in opposite directions. The two O atoms of the eth­oxy­carbonyl group are involved in intra­molecular N—H⋯O and C—H⋯O hydrogen bonds. In the crystal, weak C—H⋯O hydrogen bonds link mol­ecules related by translation along the *b* axis into chains.

## Related literature

For applications of functionalized piperidines, see: Viegas *et al.* (2004 ▶); Kobayashi *et al.* (1999 ▶). For the crystal structures of related compounds, see: Khan *et al.* (2008 ▶); Brahmachari & Das (2012 ▶).
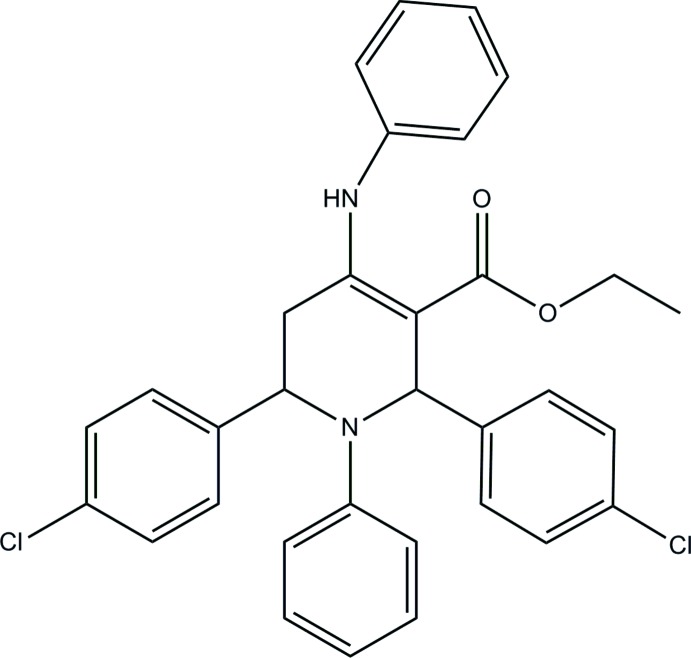


## Experimental

### 

#### Crystal data

C_32_H_28_Cl_2_N_2_O_2_
*M* *_r_* = 543.46
Triclinic, 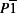

*a* = 9.559 (4) Å
*b* = 9.656 (3) Å
*c* = 16.392 (6) Å
α = 78.584 (6)°
β = 82.056 (6)°
γ = 68.390 (6)°
*V* = 1375.3 (8) Å^3^
*Z* = 2
Mo *K*α radiation
μ = 0.27 mm^−1^
*T* = 296 K
0.26 × 0.22 × 0.19 mm

#### Data collection

Bruker SMART CCD area-detector diffractometer
Absorption correction: multi-scan (*SADABS*; Sheldrick, 1996 ▶) *T*_min_ = 0.934, *T*_max_ = 0.951
6863 measured reflections
4767 independent reflections
3177 reflections with *I* > 2σ(*I*)
*R*_int_ = 0.022

#### Refinement

*R*[*F* ^2^ > 2σ(*F* ^2^)] = 0.070
*wR*(*F* ^2^) = 0.310
*S* = 1.08
4767 reflections
344 parameters
H-atom parameters constrained
Δρ_max_ = 0.49 e Å^−3^
Δρ_min_ = −0.47 e Å^−3^

### 

Data collection: *SMART* (Bruker, 2007 ▶); cell refinement: *SAINT* (Bruker, 2007 ▶); data reduction: *SAINT*; program(s) used to solve structure: *SHELXS97* (Sheldrick, 2008 ▶); program(s) used to refine structure: *SHELXL97* (Sheldrick, 2008 ▶); molecular graphics: *SHELXTL* (Sheldrick, 2008 ▶); software used to prepare material for publication: *SHELXTL*.

## Supplementary Material

Crystal structure: contains datablock(s) I, global. DOI: 10.1107/S1600536813013573/cv5409sup1.cif

Structure factors: contains datablock(s) I. DOI: 10.1107/S1600536813013573/cv5409Isup2.hkl

Supplementary material file. DOI: 10.1107/S1600536813013573/cv5409Isup3.cml

Additional supplementary materials:  crystallographic information; 3D view; checkCIF report

## Figures and Tables

**Table 1:** Hydrogen-bond geometry (Å, °)

*D*—H⋯*A*	*D*—H	H⋯*A*	*D*⋯*A*	*D*—H⋯*A*
N1—H1⋯O1	0.86	2.02	2.659 (5)	130
C4—H4⋯O2	0.98	2.30	2.761 (5)	108
C22—H22⋯O1^i^	0.93	2.69	3.287 (6)	122

Symmetry code: (i) 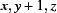.

**Fig. 1.**
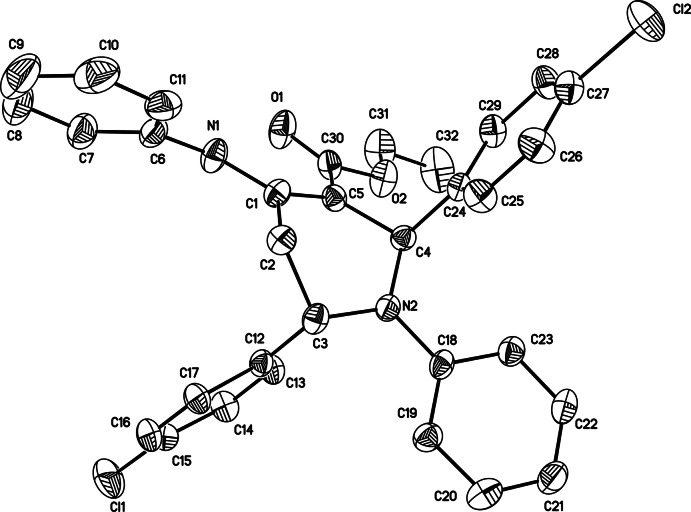
The molecular structure of the title compound showing the atomic numbering and 30% probabilty displacement ellipsoids. H atoms omitted for clarity.

## supplementary crystallographic information

### Comment

Functionalized piperidines occur with great regularity in the natural product arena and as important units in pharmaceuticals (Viegas *et al.*, 2004; Kobayashi *et al.*, 1999). As a continuation of structural study of functionalized piperidines (Brahmachari *et al.*, 2012; Khan *et al.*, 2008), herewith we present the title compound.

In (I) (Fig. 1), the bond lengths and angles are normal and correspond to those observed in anti-ethyl 4-anilino-1,2,6-triphenyl-1,2,5,6-tetrahydro-3- pyridinecarboxylate and ethyl 4-anilino-1,2,6-triphenyl-1,2,5,6-tetrahydropyridine-3-carboxylate (Khan *et al.*, 2008; Brahmachari *et al.*, 2012). The molecule of (I) contains one 1,2,5,6-tetrahydropyridine ring, ester group and four benzene rings. The central 1,2,5,6-tetrahydropyridine ring exhibits a distorted boat conformation, with the benzene rings attached to the C1, C3, N2 and C4 atoms. Two *p*-chorobenzene groups lie in the *trans*- position of the boat. The dihedral angles between the phenyl ring C6—C11 and phenyl rings C12—C17, C18—C23 and C24—C29 are 39.87 (1)°,85.04 (1)° and 65.60 (1)°, respectively. Two O atoms of the ethoxycarbonyl group are involved in intramolecular hydrogen bonds, N—H···O and C—H···O (Table 1), respectively.

In the crystal, weak intermolecular C—H···O hydrogen bonds (Table 1) link the molecules related by translation along the *b* axis into chains.

### Experimental

A 50 ml flask was charged with a magnetic stir bar, *p*-chlorobenzaldehyde (5 mmol), ethylacetoacetate (2.5 mmol) and bismuth nitrate (0.005 mmol) in 10 ml e thanol; the mixture was then started to stir at room temperature. After 1 h min, *p*-chlorobenzaldehyde (5 mmol) was added to the reaction mixture and stirring was continued up to completion of the reaction as monitored by TLC. After completion of the reaction, a thick precipitate was obtained. The solid was dissolved in hot ethyl acetate-ethanol mixture, the filtrate on standing afforded crystals of the pure product.

### Refinement

All H atoms were placed in geometrically idealized positions (C—H 0.93–0.97 Å, N—H 0.86 Å) and treated as riding on their parent atoms, with *U*_iso_(H) = 1.2–1.5*U*_eq_(C, N).

### Crystal data

**Table:**

C_32_H_28_Cl_2_N_2_O_2_	*Z* = 2
*M**_r_* = 543.46	*F*(000) = 568
Triclinic, *P*1	*D*_x_ = 1.312 Mg m^−^^3^
*a* = 9.559 (4) Å	Mo *K*α radiation, λ = 0.71073 Å
*b* = 9.656 (3) Å	Cell parameters from 1798 reflections
*c* = 16.392 (6) Å	θ = 2.5–25.5°
α = 78.584 (6)°	µ = 0.27 mm^−^^1^
β = 82.056 (6)°	*T* = 296 K
γ = 68.390 (6)°	Block, yellow
*V* = 1375.3 (8) Å^3^	0.26 × 0.22 × 0.19 mm

### Data collection

**Table:**

Bruker SMART CCD area-detector diffractometer	4767 independent reflections
Radiation source: fine-focus sealed tube	3177 reflections with *I* > 2σ(*I*)
Graphite monochromator	*R*_int_ = 0.022
phi and ω scans	θ_max_ = 25.1°, θ_min_ = 2.3°
Absorption correction: multi-scan (*SADABS*; Sheldrick, 1996)	*h* = −11→11
*T*_min_ = 0.934, *T*_max_ = 0.951	*k* = −11→8
6863 measured reflections	*l* = −19→15

### Refinement

**Table:**

Refinement on *F*^2^	Primary atom site location: structure-invariant direct methods
Least-squares matrix: full	Secondary atom site location: difference Fourier map
*R*[*F*^2^ > 2σ(*F*^2^)] = 0.070	Hydrogen site location: inferred from neighbouring sites
*wR*(*F*^2^) = 0.310	H-atom parameters constrained
*S* = 1.08	*w* = 1/[σ^2^(*F*_o_^2^) + (0.2*P*)^2^ + 0.5326*P*] where *P* = (*F*_o_^2^ + 2*F*_c_^2^)/3
4767 reflections	(Δ/σ)_max_ = 0.019
344 parameters	Δρ_max_ = 0.49 e Å^−^^3^
0 restraints	Δρ_min_ = −0.47 e Å^−^^3^

### Special details

**Table:**

Geometry. All e.s.d.'s (except the e.s.d. in the dihedral angle between two l.s. planes) are estimated using the full covariance matrix. The cell e.s.d.'s are taken into account individually in the estimation of e.s.d.'s in distances, angles and torsion angles; correlations between e.s.d.'s in cell parameters are only used when they are defined by crystal symmetry. An approximate (isotropic) treatment of cell e.s.d.'s is used for estimating e.s.d.'s involving l.s. planes.
Refinement. Refinement of *F*^2^ against ALL reflections. The weighted *R*-factor *wR* and goodness of fit *S* are based on *F*^2^, conventional *R*-factors *R* are based on *F*, with *F* set to zero for negative *F*^2^. The threshold expression of *F*^2^ > σ(*F*^2^) is used only for calculating *R*-factors(gt) *etc*. and is not relevant to the choice of reflections for refinement. *R*-factors based on *F*^2^ are statistically about twice as large as those based on *F*, and *R*- factors based on ALL data will be even larger.

### Fractional atomic coordinates and isotropic or equivalent isotropic displacement parameters (Å^2^)

**Table:**

	*x*	*y*	*z*	*U*_iso_*/*U*_eq_	
Cl1	1.27799 (17)	0.38291 (18)	0.06256 (10)	0.0849 (6)	
Cl2	0.09798 (15)	1.31946 (16)	0.46084 (8)	0.0691 (5)	
N1	0.6407 (5)	0.5253 (4)	0.3247 (2)	0.0506 (9)	
H1	0.6027	0.4977	0.2889	0.061*	
N2	0.7215 (4)	0.9294 (4)	0.2401 (2)	0.0402 (8)	
O1	0.4493 (4)	0.6217 (4)	0.2051 (2)	0.0678 (10)	
O2	0.4070 (4)	0.8552 (4)	0.1351 (2)	0.0603 (9)	
C1	0.6425 (5)	0.6679 (5)	0.3057 (3)	0.0419 (10)	
C2	0.7482 (5)	0.7082 (5)	0.3480 (2)	0.0421 (9)	
H2A	0.8212	0.6170	0.3754	0.051*	
H2B	0.6920	0.7693	0.3902	0.051*	
C3	0.8308 (5)	0.7960 (4)	0.2840 (2)	0.0405 (9)	
H3	0.8882	0.8310	0.3152	0.049*	
C4	0.5689 (4)	0.9325 (4)	0.2344 (2)	0.0371 (9)	
H4	0.5410	0.9801	0.1775	0.045*	
C5	0.5638 (4)	0.7749 (4)	0.2465 (2)	0.0388 (9)	
C6	0.6926 (5)	0.4163 (5)	0.3953 (3)	0.0494 (11)	
C7	0.7525 (6)	0.2653 (5)	0.3857 (4)	0.0641 (13)	
H7	0.7638	0.2389	0.3330	0.077*	
C8	0.7951 (8)	0.1552 (7)	0.4538 (5)	0.088 (2)	
H8	0.8324	0.0542	0.4472	0.106*	
C9	0.7834 (9)	0.1921 (9)	0.5310 (5)	0.099 (3)	
H9	0.8170	0.1170	0.5764	0.119*	
C10	0.7204 (7)	0.3435 (8)	0.5415 (4)	0.0806 (18)	
H10	0.7077	0.3691	0.5945	0.097*	
C11	0.6774 (6)	0.4544 (6)	0.4736 (3)	0.0603 (13)	
H11	0.6379	0.5553	0.4804	0.072*	
C12	0.9429 (4)	0.6958 (4)	0.2266 (2)	0.0382 (9)	
C13	0.9158 (5)	0.7039 (5)	0.1448 (3)	0.0454 (10)	
H13	0.8267	0.7731	0.1235	0.054*	
C14	1.0212 (5)	0.6088 (5)	0.0943 (3)	0.0516 (11)	
H14	1.0037	0.6157	0.0390	0.062*	
C15	1.1500 (5)	0.5056 (5)	0.1261 (3)	0.0503 (11)	
C16	1.1801 (5)	0.4959 (5)	0.2059 (3)	0.0584 (13)	
H16	1.2691	0.4253	0.2267	0.070*	
C17	1.0788 (5)	0.5906 (5)	0.2554 (3)	0.0469 (10)	
H17	1.1007	0.5850	0.3098	0.056*	
C18	0.7597 (5)	1.0556 (4)	0.2053 (2)	0.0403 (9)	
C19	0.9054 (5)	1.0534 (5)	0.2023 (3)	0.0464 (10)	
H19	0.9813	0.9653	0.2230	0.056*	
C20	0.9399 (6)	1.1826 (6)	0.1684 (3)	0.0581 (12)	
H20	1.0386	1.1794	0.1675	0.070*	
C21	0.8328 (6)	1.3126 (5)	0.1367 (3)	0.0603 (13)	
H21	0.8570	1.3980	0.1146	0.072*	
C22	0.6888 (6)	1.3157 (5)	0.1378 (3)	0.0541 (12)	
H22	0.6148	1.4036	0.1151	0.065*	
C23	0.6513 (5)	1.1904 (5)	0.1721 (3)	0.0463 (10)	
H23	0.5519	1.1959	0.1731	0.056*	
C24	0.4517 (4)	1.0286 (4)	0.2940 (2)	0.0362 (9)	
C25	0.4924 (5)	1.0732 (5)	0.3592 (3)	0.0504 (11)	
H25	0.5940	1.0438	0.3683	0.060*	
C26	0.3851 (5)	1.1606 (6)	0.4112 (3)	0.0552 (12)	
H26	0.4137	1.1895	0.4552	0.066*	
C27	0.2356 (5)	1.2047 (5)	0.3973 (3)	0.0472 (10)	
C28	0.1902 (5)	1.1639 (5)	0.3319 (3)	0.0502 (11)	
H28	0.0886	1.1953	0.3223	0.060*	
C29	0.2995 (5)	1.0758 (5)	0.2818 (3)	0.0462 (10)	
H29	0.2705	1.0466	0.2379	0.055*	
C30	0.4708 (5)	0.7404 (5)	0.1958 (3)	0.0453 (10)	
C31	0.3141 (7)	0.8271 (7)	0.0817 (4)	0.0794 (18)	
H31A	0.2309	0.8039	0.1148	0.095*	
H31B	0.3736	0.7420	0.0538	0.095*	
C32	0.2570 (9)	0.9630 (8)	0.0205 (4)	0.096 (2)	
H32A	0.1894	1.0437	0.0483	0.145*	
H32B	0.2042	0.9438	−0.0193	0.145*	
H32C	0.3399	0.9908	−0.0079	0.145*	

### Atomic displacement parameters (Å^2^)

**Table:**

	*U*^11^	*U*^22^	*U*^33^	*U*^12^	*U*^13^	*U*^23^
Cl1	0.0700 (10)	0.0711 (10)	0.0914 (11)	0.0050 (7)	0.0067 (8)	−0.0297 (8)
Cl2	0.0595 (8)	0.0782 (9)	0.0611 (8)	−0.0053 (6)	0.0006 (6)	−0.0325 (7)
N1	0.065 (2)	0.0365 (19)	0.056 (2)	−0.0222 (17)	−0.0190 (18)	−0.0033 (16)
N2	0.0390 (18)	0.0324 (17)	0.049 (2)	−0.0133 (14)	−0.0061 (15)	−0.0038 (14)
O1	0.076 (2)	0.0438 (19)	0.095 (3)	−0.0248 (17)	−0.043 (2)	−0.0047 (18)
O2	0.079 (2)	0.0481 (18)	0.059 (2)	−0.0205 (17)	−0.0343 (17)	−0.0056 (15)
C1	0.049 (2)	0.037 (2)	0.044 (2)	−0.0191 (18)	−0.0037 (18)	−0.0065 (17)
C2	0.045 (2)	0.044 (2)	0.039 (2)	−0.0160 (19)	−0.0061 (17)	−0.0052 (17)
C3	0.050 (2)	0.035 (2)	0.041 (2)	−0.0171 (18)	−0.0121 (18)	−0.0060 (17)
C4	0.037 (2)	0.037 (2)	0.039 (2)	−0.0149 (17)	−0.0053 (16)	−0.0046 (16)
C5	0.038 (2)	0.036 (2)	0.044 (2)	−0.0111 (17)	−0.0048 (17)	−0.0114 (17)
C6	0.050 (3)	0.041 (2)	0.059 (3)	−0.023 (2)	−0.011 (2)	0.005 (2)
C7	0.069 (3)	0.046 (3)	0.080 (4)	−0.023 (2)	−0.012 (3)	−0.004 (2)
C8	0.085 (4)	0.051 (3)	0.126 (6)	−0.028 (3)	−0.035 (4)	0.018 (4)
C9	0.106 (5)	0.088 (5)	0.105 (6)	−0.053 (4)	−0.041 (4)	0.044 (4)
C10	0.080 (4)	0.103 (5)	0.062 (3)	−0.047 (4)	−0.009 (3)	0.011 (3)
C11	0.057 (3)	0.068 (3)	0.053 (3)	−0.026 (3)	0.007 (2)	−0.003 (2)
C12	0.038 (2)	0.031 (2)	0.044 (2)	−0.0117 (17)	−0.0122 (16)	0.0014 (16)
C13	0.044 (2)	0.041 (2)	0.045 (2)	−0.0073 (18)	−0.0086 (18)	−0.0044 (18)
C14	0.052 (3)	0.051 (3)	0.047 (2)	−0.012 (2)	−0.007 (2)	−0.010 (2)
C15	0.044 (2)	0.040 (2)	0.064 (3)	−0.014 (2)	−0.001 (2)	−0.006 (2)
C16	0.043 (3)	0.045 (3)	0.074 (3)	−0.003 (2)	−0.017 (2)	0.006 (2)
C17	0.037 (2)	0.045 (2)	0.053 (2)	−0.0064 (19)	−0.0157 (18)	0.0011 (19)
C18	0.054 (2)	0.032 (2)	0.039 (2)	−0.0166 (18)	0.0011 (17)	−0.0121 (16)
C19	0.049 (2)	0.045 (2)	0.050 (2)	−0.021 (2)	−0.0018 (19)	−0.0106 (19)
C20	0.069 (3)	0.062 (3)	0.054 (3)	−0.038 (3)	−0.006 (2)	−0.006 (2)
C21	0.071 (3)	0.046 (3)	0.067 (3)	−0.028 (3)	−0.001 (3)	−0.004 (2)
C22	0.072 (3)	0.036 (2)	0.053 (3)	−0.017 (2)	−0.005 (2)	−0.007 (2)
C23	0.054 (3)	0.040 (2)	0.045 (2)	−0.017 (2)	−0.0001 (19)	−0.0088 (18)
C24	0.042 (2)	0.0295 (19)	0.038 (2)	−0.0125 (16)	−0.0084 (16)	−0.0028 (15)
C25	0.046 (2)	0.058 (3)	0.052 (2)	−0.017 (2)	−0.0110 (19)	−0.016 (2)
C26	0.055 (3)	0.069 (3)	0.048 (3)	−0.021 (2)	−0.002 (2)	−0.027 (2)
C27	0.053 (3)	0.043 (2)	0.044 (2)	−0.013 (2)	−0.0071 (19)	−0.0077 (18)
C28	0.040 (2)	0.048 (3)	0.058 (3)	−0.005 (2)	−0.013 (2)	−0.011 (2)
C29	0.050 (2)	0.041 (2)	0.050 (2)	−0.0119 (19)	−0.0154 (19)	−0.0117 (19)
C30	0.042 (2)	0.037 (2)	0.056 (3)	−0.0080 (18)	−0.0125 (19)	−0.0126 (19)
C31	0.094 (4)	0.069 (4)	0.081 (4)	−0.018 (3)	−0.046 (3)	−0.018 (3)
C32	0.119 (6)	0.091 (5)	0.070 (4)	−0.010 (4)	−0.045 (4)	−0.020 (3)

### Geometric parameters (Å, º)

**Table:**

Cl1—C15	1.747 (5)	C13—C14	1.390 (6)
Cl2—C27	1.746 (5)	C13—H13	0.9300
N1—C1	1.356 (5)	C14—C15	1.360 (6)
N1—C6	1.410 (6)	C14—H14	0.9300
N1—H1	0.8600	C15—C16	1.356 (7)
N2—C18	1.393 (5)	C16—C17	1.363 (7)
N2—C3	1.455 (5)	C16—H16	0.9300
N2—C4	1.463 (5)	C17—H17	0.9300
O1—C30	1.215 (5)	C18—C19	1.379 (6)
O2—C30	1.350 (5)	C18—C23	1.395 (6)
O2—C31	1.454 (6)	C19—C20	1.397 (6)
C1—C5	1.344 (6)	C19—H19	0.9300
C1—C2	1.499 (6)	C20—C21	1.355 (7)
C2—C3	1.534 (6)	C20—H20	0.9300
C2—H2A	0.9700	C21—C22	1.363 (7)
C2—H2B	0.9700	C21—H21	0.9300
C3—C12	1.513 (6)	C22—C23	1.379 (6)
C3—H3	0.9800	C22—H22	0.9300
C4—C5	1.513 (5)	C23—H23	0.9300
C4—C24	1.537 (5)	C24—C25	1.379 (6)
C4—H4	0.9800	C24—C29	1.386 (6)
C5—C30	1.458 (6)	C25—C26	1.377 (6)
C6—C11	1.378 (7)	C25—H25	0.9300
C6—C7	1.389 (7)	C26—C27	1.370 (6)
C7—C8	1.371 (8)	C26—H26	0.9300
C7—H7	0.9300	C27—C28	1.384 (6)
C8—C9	1.359 (11)	C28—C29	1.370 (6)
C8—H8	0.9300	C28—H28	0.9300
C9—C10	1.397 (10)	C29—H29	0.9300
C9—H9	0.9300	C31—C32	1.460 (8)
C10—C11	1.373 (8)	C31—H31A	0.9700
C10—H10	0.9300	C31—H31B	0.9700
C11—H11	0.9300	C32—H32A	0.9600
C12—C13	1.382 (6)	C32—H32B	0.9600
C12—C17	1.397 (5)	C32—H32C	0.9600
			
C1—N1—C6	128.7 (4)	C16—C15—Cl1	119.8 (4)
C1—N1—H1	115.7	C14—C15—Cl1	119.0 (4)
C6—N1—H1	115.7	C15—C16—C17	119.4 (4)
C18—N2—C3	120.5 (3)	C15—C16—H16	120.3
C18—N2—C4	119.7 (3)	C17—C16—H16	120.3
C3—N2—C4	119.8 (3)	C16—C17—C12	121.6 (4)
C30—O2—C31	116.2 (4)	C16—C17—H17	119.2
C5—C1—N1	124.8 (4)	C12—C17—H17	119.2
C5—C1—C2	116.4 (3)	C19—C18—N2	121.8 (4)
N1—C1—C2	118.7 (4)	C19—C18—C23	117.1 (4)
C1—C2—C3	110.2 (3)	N2—C18—C23	121.1 (4)
C1—C2—H2A	109.6	C18—C19—C20	120.5 (4)
C3—C2—H2A	109.6	C18—C19—H19	119.7
C1—C2—H2B	109.6	C20—C19—H19	119.7
C3—C2—H2B	109.6	C21—C20—C19	121.4 (5)
H2A—C2—H2B	108.1	C21—C20—H20	119.3
N2—C3—C12	113.6 (3)	C19—C20—H20	119.3
N2—C3—C2	109.8 (3)	C20—C21—C22	118.7 (4)
C12—C3—C2	111.5 (3)	C20—C21—H21	120.6
N2—C3—H3	107.2	C22—C21—H21	120.6
C12—C3—H3	107.2	C21—C22—C23	121.0 (5)
C2—C3—H3	107.2	C21—C22—H22	119.5
N2—C4—C5	111.6 (3)	C23—C22—H22	119.5
N2—C4—C24	112.6 (3)	C22—C23—C18	121.2 (4)
C5—C4—C24	111.3 (3)	C22—C23—H23	119.4
N2—C4—H4	107.0	C18—C23—H23	119.4
C5—C4—H4	107.0	C25—C24—C29	118.0 (4)
C24—C4—H4	107.0	C25—C24—C4	122.2 (3)
C1—C5—C30	120.2 (4)	C29—C24—C4	119.8 (3)
C1—C5—C4	119.7 (3)	C26—C25—C24	121.0 (4)
C30—C5—C4	120.1 (3)	C26—C25—H25	119.5
C11—C6—C7	119.5 (4)	C24—C25—H25	119.5
C11—C6—N1	122.1 (4)	C27—C26—C25	119.4 (4)
C7—C6—N1	118.3 (5)	C27—C26—H26	120.3
C8—C7—C6	120.1 (6)	C25—C26—H26	120.3
C8—C7—H7	120.0	C26—C27—C28	121.4 (4)
C6—C7—H7	120.0	C26—C27—Cl2	120.1 (3)
C9—C8—C7	120.7 (6)	C28—C27—Cl2	118.5 (3)
C9—C8—H8	119.7	C29—C28—C27	117.9 (4)
C7—C8—H8	119.7	C29—C28—H28	121.1
C8—C9—C10	119.6 (6)	C27—C28—H28	121.1
C8—C9—H9	120.2	C28—C29—C24	122.4 (4)
C10—C9—H9	120.2	C28—C29—H29	118.8
C11—C10—C9	120.0 (6)	C24—C29—H29	118.8
C11—C10—H10	120.0	O1—C30—O2	121.2 (4)
C9—C10—H10	120.0	O1—C30—C5	125.2 (4)
C10—C11—C6	120.0 (6)	O2—C30—C5	113.6 (4)
C10—C11—H11	120.0	O2—C31—C32	107.8 (5)
C6—C11—H11	120.0	O2—C31—H31A	110.1
C13—C12—C17	117.7 (4)	C32—C31—H31A	110.2
C13—C12—C3	122.1 (3)	O2—C31—H31B	110.2
C17—C12—C3	120.3 (4)	C32—C31—H31B	110.1
C12—C13—C14	120.2 (4)	H31A—C31—H31B	108.5
C12—C13—H13	119.9	C31—C32—H32A	109.5
C14—C13—H13	119.9	C31—C32—H32B	109.5
C15—C14—C13	119.8 (4)	H32A—C32—H32B	109.5
C15—C14—H14	120.1	C31—C32—H32C	109.5
C13—C14—H14	120.1	H32A—C32—H32C	109.5
C16—C15—C14	121.3 (4)	H32B—C32—H32C	109.5

### Hydrogen-bond geometry (Å, º)

**Table:**

*D*—H···*A*	*D*—H	H···*A*	*D*···*A*	*D*—H···*A*
N1—H1···O1	0.86	2.02	2.659 (5)	130
C4—H4···O2	0.98	2.30	2.761 (5)	108
C22—H22···O1^i^	0.93	2.69	3.287 (6)	122

Symmetry code: (i) *x*, *y*+1, *z*.
